# Supplementary material for: Factors associated with work engagement of nurses in the radiology department, Japan: a cross-sectional study
Source: PeerJ. 2024 Nov 18;12:e18426. doi: 10.7717/peerj.18426 (PMC11580675; doi:10.7717/peerj.18426)
Supplement: Supplemental Information 2 [file peerj-12-18426-s002.docx]

1. The following 17 statements are about how you feel at work. Please read each statement carefully and decide if you ever feel this way about your job.
2. At my work, I feel bursting with energy

　
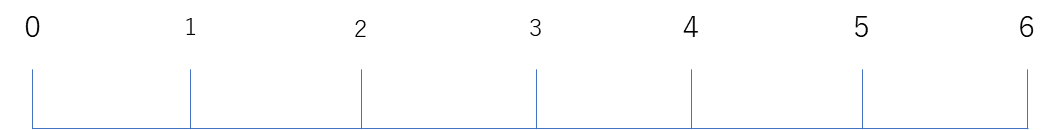


Everyday

Never

1. I find the work that I do full of meaning and purpose

　
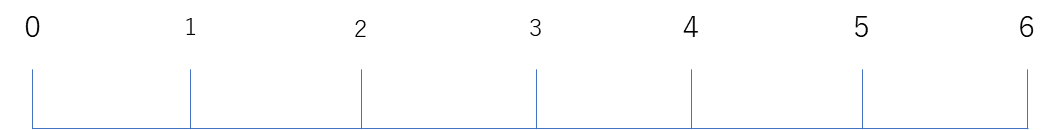


Never

Everyday

1. Time flies when I'm working

　
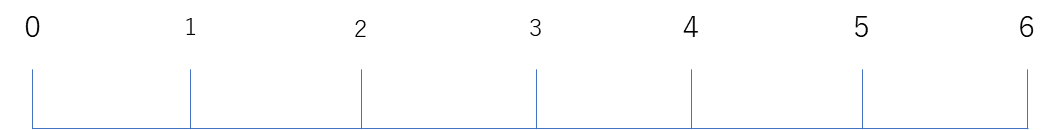


Never

Everyday

1. At my job, I feel strong and vigorous

　
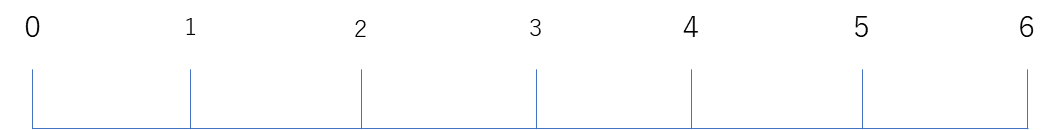


Everyday

Never

1. I am enthusiastic about my job

　
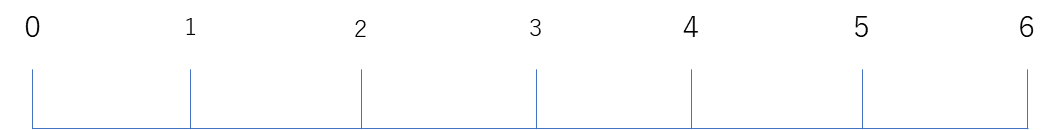


Everyday

Never

1. When I am working, I forget everything else around me

　
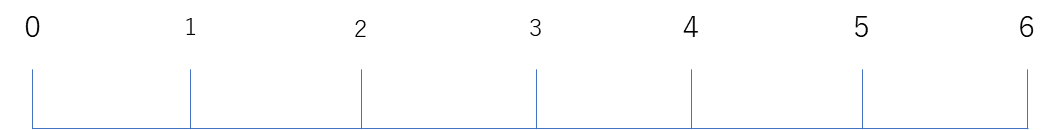


Never

Everyday

1. My job inspires me

　
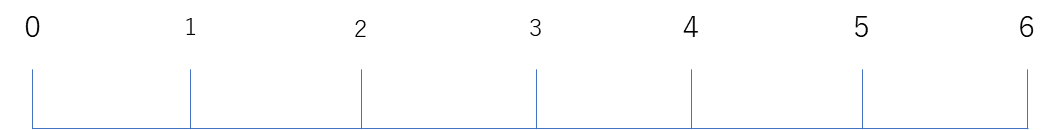


Never

Everyday

1. When I get up in the morning, I feel like going to work

　
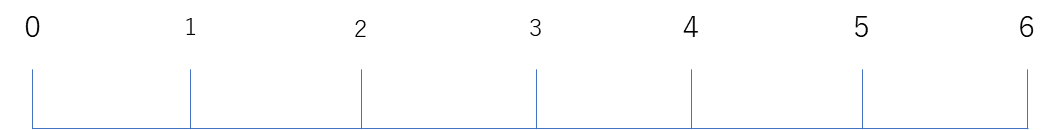


Everyday

Never

1. I feel happy when I am working intensely

　
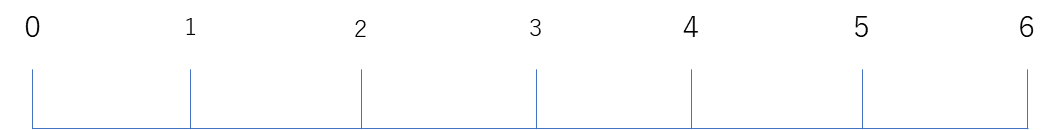


Never

Everyday

1. I am proud on the work that I do

　
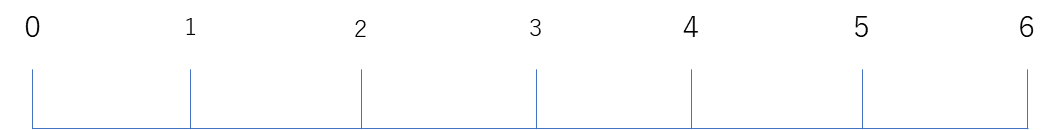


Never

Everyday

1. I am immersed in my work

　
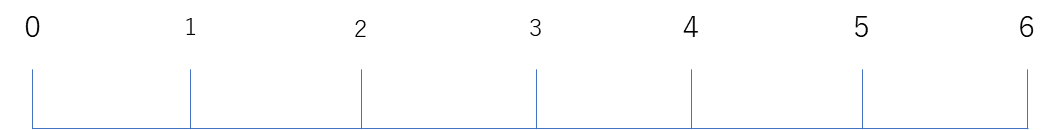


Never

Everyday

1. I can continue working for very long periods at a time

　
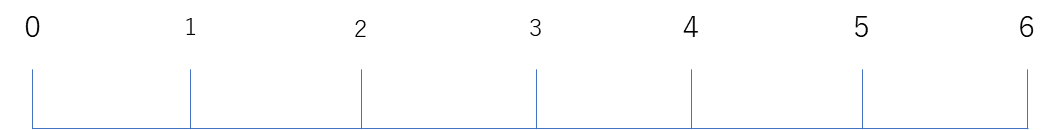


Everyday

Never

1. To me, my job is challenging

　
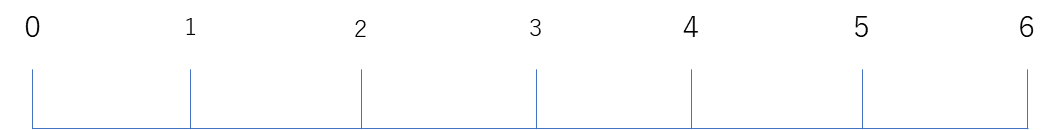


Never

Everyday

1. I get carried away when I’m working

　
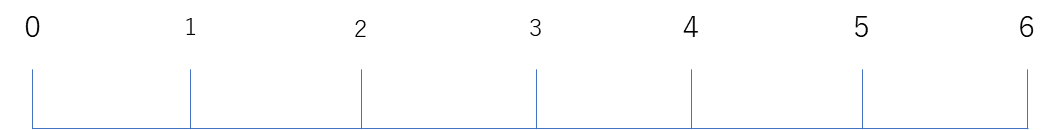


Never

Everyday

1. At my job, I am very resilient, mentally

　
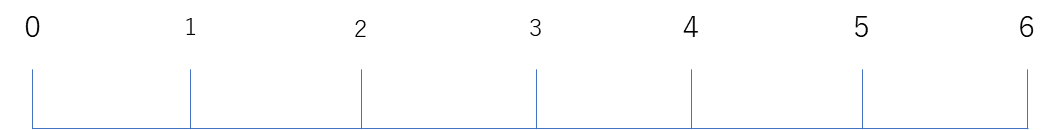


Everyday

Never

1. It is difficult to detach myself from my job

　
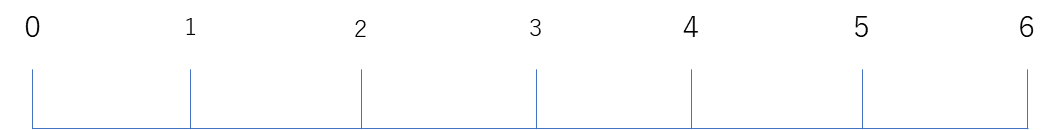


Never

Everyday

1. At my work I always persevere, even when things do not go well

　
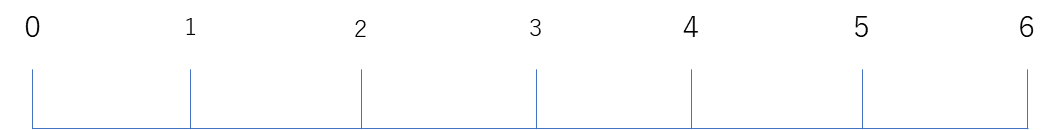


Everyday

Never

1. Please tell me how you feel about working in the field of radiology.
2. How do you feel about the dose on your personal dosimeter?

　
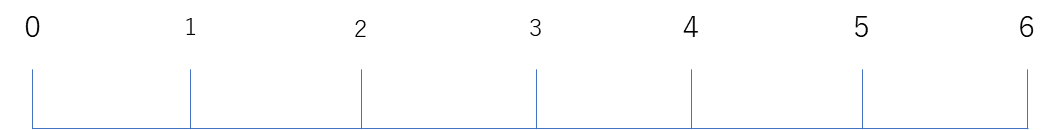


Very much

Very little

1. Is radiation exposure management being carried out appropriately at your workplace?　
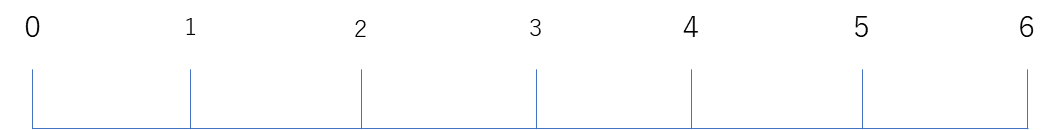


Not at all

Very much so

1. Does your workplace have a system in place for consultation about exposure to radiation?
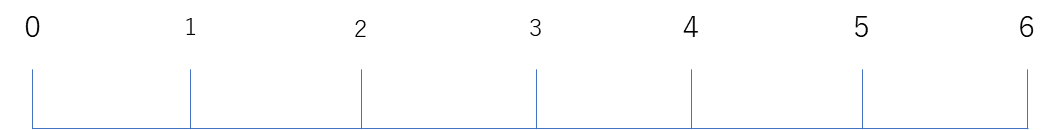


Very much so

Not at all

1.
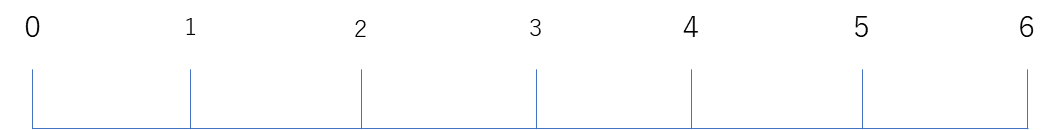
Do you think your radiation protection measures are sufficient?

Not at all

Very much so

1.
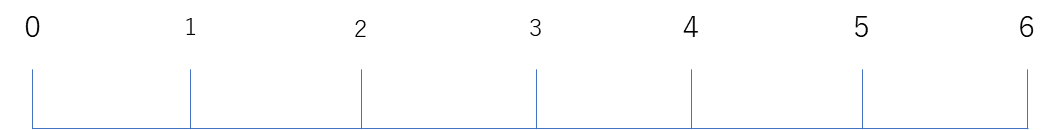
Do you think there is an effect on your health from occupational radiation exposure?

Very much so

Not at all

1.
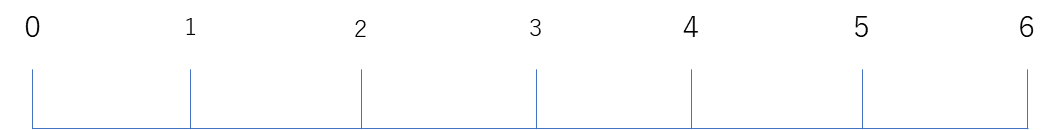
Do you think your radiation exposure will affect the health of your children?

Not at all

Very much so

1. Please tell me about yourself
2. Age: (　　　) years old
3. Gender: Female, Male
4. Marital status: Married, Unmarried
5. Spouse: Yes, No
6. Presence of children under 18 years of age: Yes, No
7. Years of nursing experience: < 5 years, 5-10 years, 11-15 years, 16-20 years, 21-25 years, 26-30 years, 31 years <
8. Years of departmental experience: ( ) years
9. Position: Staff nurse, Manager nurse
10. Post-employment qualifications: Yes, No
11. Desire to be assigned to the radiology department: Yes, No
12. Current main nursing operations (multiple answers allowed): Assistance in diagnostic imaging, Angiography, Endoscopy/Fluoroscopy, Nuclear medicine, Radiation therapy, Other
